# No assembly required: Time for stronger, simpler publishing standards for DNA sequences

**DOI:** 10.1371/journal.pbio.3002376

**Published:** 2023-11-16

**Authors:** B W. Thuronyi, Erika A. DeBenedictis, Jeffrey E. Barrick

**Affiliations:** 1 Department of Chemistry, Williams College, Williamstown, Massachusetts, United States of America; 2 Biodesign Laboratory, Francis Crick Institute, London, United Kingdom; 3 Department of Molecular Biosciences, Center for Systems and Synthetic Biology, The University of Texas at Austin, Austin, Texas, United States of America

## Abstract

Describing how DNA constructs were assembled is no longer necessary when it is possible to fully sequence the final results to validate them. In this Perspective, the authors suggest that outdated methods narratives should be replaced with DNA sequence files when publishing life sciences research.

## Teaser

Uniformly accessible DNA sequences are needed to improve experimental reproducibility and automation. Rather than descriptions of how engineered DNA is assembled, publishers should require complete and empirically validated sequences.

Every scientific discipline develops standards for publishing that are designed to help researchers succinctly communicate evidence supporting their conclusions and allow others to build upon their work. For example, to publish the first report of a new compound, synthetic chemists must supply NMR and mass spectra because there is a broad consensus that these analytical techniques are necessary to show that a compound was prepared as intended. Similarly, when new software is developed, it is nearly universally required that the source code be made available upon publication so others can check its function and reuse it.

In 2011, it was proposed that full DNA sequences should be reported to support synthetic biology publications [1]. Yet today bioengineering and other areas of the life sciences still suffer from confusing, inconsistent, and insufficient standards for publishing DNA sequences. The sequences of plasmids and genomes developed during a study are sometimes not included as part of a publication at all or only in the form of instructions for how to assemble them instead of the final sequences. This would be akin to a computer science paper omitting its code or describing how the code could be reconstructed by copying snippets from code in other papers. If biologists are reprogramming life, why are they not expected to publish their source code?

Some practices are holdovers from when engineered DNA was constructed by copying and pasting together parts of existing DNA sequences and it was expensive, difficult, or even impossible to check the results. However, sequencing technologies have now improved to the point that determining the entire nucleotide sequences of plasmids and even genomes is becoming inexpensive and widely accessible. In 2023, sequencing an entire plasmid costs $15 and sequencing a bacterial genome costs $100, and these prices are likely to fall. Unlike computer code, DNA can mutate, accumulating changes in its sequence when it is copied by enzymes or cells. This possibility of unintentional evolution makes it especially important to verify DNA sequences being used in a study, even if they are not newly constructed.

DNA synthesis technologies are also improving. It is increasingly becoming possible and affordable to order plasmids and large pieces of DNA that are constructed from scratch. We are moving toward an era in which most new DNA constructs will be made on demand by biomanufacturing companies, rather than painstakingly assembled from existing pieces by the researchers who designed them. These changes negate the utility of describing the assembly process in a publication, since DNA will function the same way no matter how it is made.

In light of these developments, we suggest new standards and best practices for publishing life sciences research that uses engineered DNA constructs (Box 1).

**Box 1. Proposed publishing standards for studies that include engineered DNA sequences**

**Required**

- Report the complete sequences of all plasmids and other DNA constructs.
- Describe which parts of each DNA sequence were empirically validated and how.
- Provide DNA sequences in a nonproprietary, machine-readable, text file format. GenBank format is recommended. FASTA format is permitted.

**Recommended**

- Include author annotations of sequence features that are important for interpreting results.
- Deposit DNA sequences in public databases.
- Submit samples of engineered plasmids and cells to nonprofit repositories.

**Deprecated**

- Detailed narrative descriptions of synthesis, assembly, and/or cloning steps used to create DNA sequences, except when methods development is the focus of a study.

**Best practices example**

The study by Meyer and colleagues that developed “marionette” *Escherichia coli* strains and plasmids for inducible gene expression [2] follows most of our recommendations. The authors deposited annotated sequences for their plasmids in GenBank, and they archived samples of their plasmids and engineered bacterial strains with Addgene. They report how the sequence of the modified portion of the *E*. *coli* genome was validated, and an annotated file is provided on the Addgene website alongside empirically determined full plasmid sequences. We would have suggested just one improvement: providing sequences as machine-readable Supplementary Data files with the publication, rather than embedding them in tables in a merged Supplementary Information PDF document.

Our standards require that authors report the entire sequence of every DNA construct used in their experiments. Even if they consider some portions, such as a plasmid backbone, less important or relevant, the full sequence must be provided. Complete reporting avoids tedious reference tracking and/or guesswork to reconstruct sequences, eliminates ambiguities, and ensures that researchers have validated their materials. Combinatorial libraries that include many sequences can be exemplified by a representative sequence plus enough information that any individual member can be unambiguously reconstructed. Although we recommend the routine use of whole-genome sequencing to characterize modified cells or organisms, we recognize this remains somewhat costly and demanding. At a minimum, sequences must be reported for engineered regions of newly modified genomes.

Ideally, all DNA sequences reported in a study will be fully supported by empirical sequencing data. Regardless of how much validation was performed, we propose that authors be required to clearly document what portions of their sequences were verified and how. For example, if certain fragments generated by restriction enzyme digest of a part plasmid were not re-sequenced after assembly, this would be explicitly stated and/or annotated in the provided files. Although this reporting standard is more demanding than conventional practices, little elaboration is needed if all bases are verified empirically, such as by whole-plasmid sequencing. Blanket statements such as “all constructs were verified by Sanger sequencing” are not sufficiently specific.

We call for universal adoption of the GenBank file format for reporting DNA sequences. Though not preferred, because it only supports sequences and cannot include annotations, we would also allow the simpler FASTA file format. These are both established and widely used formats that are compatible with essentially all software tools and databases. All DNA sequences should be provided in one or more Supplementary Data archive files. Sequences should never be provided solely in the form of figures, spreadsheets, word processing documents, or PDF files, which provide less information and reuse value than machine-readable text file formats.

Accurate and meaningful annotation of functional DNA sequences, both natural and engineered, is challenging. Therefore, there is considerable value in publishing sequences with annotations curated by experts. Authors should be encouraged to include their annotations in published sequences. Annotations capture what they noted and recognized about their DNA materials and can inform interpretation of results, as well as reuse of genetic parts and modules. These annotations are often available without much additional effort and may provide useful training data for machine learning or other automated approaches for improving annotation pipelines.

In addition to making DNA sequences available as supplementary files, we recommend that authors digitally deposit them in public databases such as GenBank or the European Nucleotide Archive so that permanent entries with accession numbers are available. Physical samples can also be deposited in nonprofit repositories like Addgene (for plasmids) (RRID:SCR_002037) [3] or the American Type Culture Collection (for microbes) (RRID:SCR_001672) so that validated stocks will be stored and distributed. One benefit of archiving is the associated quality assurance checks. For example, GenBank enforces uniform formatting and metadata standards, and Addgene checks that each plasmid matches its designed sequence and reports discrepancies. Publicly archiving sequence information also facilitates data mining and reproducibility by providing a consistent interface to access and download versioned records.

Modern biotechnology studies often involve cloning hundreds of bespoke constructs and increasingly employ direct synthesis of large DNA fragments. Each laboratory favors its own subset of an enormous diversity of effective assembly techniques and an increasing variety of commercial sources of synthetic DNA. Unless a paper is reporting new DNA synthesis or assembly methods, detailed descriptions of how these steps were performed by the authors are unnecessary, as well as laborious to write, difficult to read, and unlikely to be useful to others in the future. Any potential utility of this information is obscured by the narrative text format. Reporting tables of primer sequences used exclusively for DNA assembly should also be optional. Information sharing is better addressed through providing full DNA sequences in machine-readable formats.

Accurate, complete, and reusable DNA sequence reporting is long overdue as a universal requirement for life sciences publishing, alongside other FAIR data management practices [4] such as minimum information standards for specific types of data [5]. As the costs for sequencing continue to fall, empirical validation of all DNA parts, from plasmids to genomes, must become standard practice. It is essential to establish a robust culture of sharing DNA sequences to accompany and encourage these developments through uniform standards that are comprehensive, yet not so onerous that a typical bench scientist cannot easily comply with them. Advancement of automated tools and other approaches—for functional annotation [6], database curation [7], DNA assembly [8,9], biosecurity attribution [10], and more—absolutely depend on the availability of engineered DNA sequences for data mining and will be spurred on by adopting the reporting standards and best practices we propose.
